# Grafting Halloysite Nanotubes with Amino or Carboxyl Groups onto Carbon Fiber Surface for Excellent Interfacial Properties of Silicone Resin Composites

**DOI:** 10.3390/polym10101171

**Published:** 2018-10-22

**Authors:** Xiandong Zhang, Guangshun Wu

**Affiliations:** School of Chemistry and Materials Science, Ludong University, Yantai 264025, China; zxdldhg@163.com

**Keywords:** carbon fibers, surface grafting, halloysite nanotubes, polymer composites, interface

## Abstract

The quality of interphase in carbon fibers (CFs) composites makes a key contribution to overall performance of composites. Here, we achieved for the first time the chemical grafting of halloysite nanotubes (HNTs) with amino or carboxyl groups onto the CFs surface aiming to increase composites interfacial strength. HNTs were grafted using 3-aminopropyltriethoxysilane (APS) followed by succinic anhydride treatment, and HNTs with amino groups (HNT–NH_2_) or carboxyl groups (HNT–COOH) were separately introduced into the interphase of composites. Functional groups of HNTs and fiber surface structures were characterized, which confirmed the modification success. The wettability between the modified CFs and resin have been enhanced obviously based on the improved fiber polarity and enhanced surface roughness by the introduced two functionalized HNTs with the uniform distributions onto fiber surface. Moreover, interfacial properties and anti-hydrothermal aging behaviors of modified methylphenylsilicone resin (MPSR) composites were improved significantly, especially for HNT–COOH grafting. In addition, the interfacial reinforcement mechanisms for untreated and modified CF composites are discussed and compared.

## 1. Introduction

Carbon fibers (CFs) have become one of the ideal reinforcements for matrix resin composites because of their strong specific strength, outstanding chemical resistance and superior environmental stability [1,2,3]. The quality of interface between the fibers and matrix resin is the main element in the properties of polymer composites, and the weak quality of fiber–matrix interface leads to inferior stress transfer efficiency at the interfacial region and the dissatisfied performance of the resulting composites [4,5]. Unfortunately, untreated CF has a smooth and inert surface, which is difficult to provide desired interfacial interactions, limiting the further application of composites seriously [6,7]. Therefore, various surface treatment methods have been proposed for changing the CFs surface from chemical inert to active with the aim to improve the quality of interface [8,9,10,11,12].

Recently, many researchers have made considerable efforts to graft the surface of CFs with various active nanoparticles to change interfacial microstructure and properties of composites [13,14,15,16]. Gao [17] prepared a new hierarchical reinforcement containing octaglycidyldimethylsilyl polyhedral oligomeric silsesquioxane (POSS) and CFs with the remarkable improvement in interfacial strength of composites. Chen [18] effectively introduced polyether imide and graphene oxide into the interface between CFs and poly(ether-ether-ketone) by the sizing process. All the hierarchical CFs/active nanoparticles reinforcements help to change the wettability and surface roughness of the fibers, and thus increase interfacial adhesion and the ultimate properties of the resulting composites.

Noteworthy, HNTs are a kind of naturally clay silicate minerals with the similar nanostructures of carbon nanotubes (CNTs), which possess an inner gibbsite octahedral sheet groups (Al–OH) and external siloxane groups (Si–O–Si) [19,20]. The structure of HNTs can be expressed with the formula Al_2_Si_2_O_5_(OH)_4_·nH_2_O [21]. As environment-friendly 1D natural nanofillers, the large surface area, high aspect ratio and the unique hollow nano-tubular structure of HNTs endow high mechanical properties and outstanding thermal stability [22]. Enormous polar groups (–OH) onto the surface of HNTs not only have a good compatibility and effective interactions with polymer resin but also can be used as the bridging sites for further functionalization of HNTs. In addition, HNTs are much cheaper than other active nanoparticles (e.g., CNTs, POSS, and graphene oxide), showing unique advantages for large-scale practical application and industrial production [23,24]. Hence, there have been great interests in preparing HNTs/polymer nanocomposites with exceptional interfacial strength, and mechanical and thermal performances of the resulting composites [25,26,27]. As everyone knows, HNTs are prone to form serious aggregation, and many modified agents have been proposed to enhance the dispersion of HNTs and interfacial properties in the system [24,28]. Yu [29] prepared dextran modified HNTs by the bridging 1,6-hexamethylene diisocyanate (HDI) and incorporated modified HNTs into polyethersulfone ultrafiltration membrane with improved antifouling property. Chao [30] reported surface modification of halloysite nanotubes with dopamine (DA) for the advanced applications. However, the bridging agents HDI or DA are very expensive. More importantly, neither agent is suitable for the preparation of hydrothermal aging resistant interface. The bridging 3-aminopropyltriethoxysilane (APS) can be easily prepared, making it much cheaper than HDI or DA. APS with both ethoxyl and amino groups not only reacts with hydroxyl groups on the surface of HNTs, but also provides massive reactive sites for further introducing active molecules onto HNTs surface. Moreover, based on the same backbone siloxane structures (Si–O) of MPSR matrix, APS molecules with the strong Si–O bond can enhance interface compatibility between HNT–NH_2_ modified CFs and MPSR matrix, and fully protect fiber–matrix interface from hydrothermal aging penetration under the harsh environment. In addition, APS is used as the conventional curing agent, which accelerates the curing process of MPSR and reduces defects, and thus increases interfacial properties of composites. Hence, surface grafting of succinic anhydride onto HNTs via the bridging agent APS has been proposed in the study. However, the preparation of modified HNTs/CF hierarchical reinforcements by chemically grafting HNTs onto the CFs surface has rarely been studied.

In this work, HNT–NH_2_ and HNT–COOH were prepared, and then separately introduced on CFs surface by chemical bonding for the first time with the aim to enhance the quality of interface and properties of composites. Surface element, functional groups and structures of HNTs, HNT–NH_2_ and HNT–COOH were characterized by Fourier transform infrared spectroscopy (FTIR), thermogravimetric analysis (TGA), and X-ray photoelectron spectroscopy (XPS). Surface morphologies and wettability of untreated and modified CFs were examined by scanning electron microscope (SEM) and dynamic contact angle analysis (DCA), respectively. Interfacial properties of MPSR composites reinforced with untreated and grafted CFs were studied systematically via interfacial shear strength (IFSS) and interlaminar shear strength (ILSS), and hydrothermal aging resistance of different composites was also evaluated. In addition, the different interfacial reinforcing mechanisms for untreated and modified CFs composites are discussed and developed.

## 2. Materials and Methods

### 2.1. Materials

CFs (3 × 10^3^ single filaments per tow, average diameter 7 μm, tensile strength 3500 MPa, and tensile modulus 230 GPa) were used as the reinforcements, and obtained from Toray Industries, Inc., Tokyo, Japan. MPSR (relative density 1.08 g·cm^−3^, molecular weight 2400 g·mol^−1^, flexural strength 308 MPa and compressive strength 150 MPa) and high-purity HNTs (purity > 98%, diameter 30–70 nm, and length 0.1–2 μm) were purchased by ShangHai Chemicals Co., Shanghai, China and Guangzhou Runwo Materials Technology Co. Ltd., Guangzhou, China, respectively. APS, succinic anhydride, and triethylamine were received from Aladdin, Shanghai, China. All other drugs and reagents, such as tetrahydrofuran (THF), 4-Dimethylaminopyridine (DMAP), toluene, lithium aluminium hydride (LiAlH_4_), *N*,*N*′-Dicyclohexyl carbodiimide (DCC), concentrated nitric acid (HNO_3_), thionyl chloride (SOCl_2_) and dimethylformamide (DMF) were purchased by Tianjin Bodi Organic Chemicals Co. Ltd., Tianjin, China.

### 2.2. Preparation of HNT–NH_2_ and HNT–COOH

**Figure 1:**
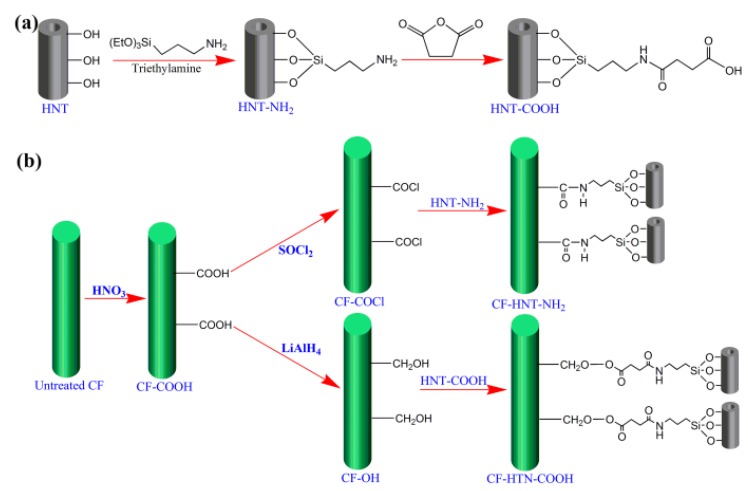
Schematic illustration of the preparation processes of: (**a**) functionalized HNTs; and (**b**) CF–HNT–NH_2_ and CF–HNT–COOH.

Surface treatment of HNTs by APS and succinic anhydride was carried out using the following procedures. Typically, HNTs (1 g) were added into the solution of 200 mL toluene by ultrasonic cleaner for 30 min. Then, 5 mL APS and the catalyst triethylamine were put into HNTs solution, and then reacted at 353 K for 24 h. After the reaction complete, the grafting of APS onto HNTs was finally centrifuged with deionized water and ethanol each for many times, obtaining HNT–NH_2_. To get HNT–COOH, 0.5 g HNT–NH_2_ nanoparticles were ultrasonically dispersed into 100 mL of 0.1 M succinic anhydride in DMF, and then reacted by stirring at room temperature for 24 h. The HNT–COOH was obtained after being washed by DMF and deionized water repeatedly and dried. Figure 1a shows the synthesis procedure of HNT–NH_2_ and HNT–COOH.

### 2.3. Functionalization of HNTs onto CFs Surface

#### 2.3.1. Fiber Desizing, Oxidation, Reduction and Acyl Chloride Modification

In a typical reaction, the pristine CFs were firstly extracted in supercritical acetone/water at 633 K for about 30 min with the aim of removing polymer sizing agents (denoted as untreated CF). Then, untreated CF was treated by concentrated HNO_3_ at 353 K for 4 h to obtain CF–COOH, which has many carboxyl functional groups. Subsequently, CF–COOH was reacted with LiAlH_4_–THF saturated solution under reflux for 2 h to introduce hydroxyl groups from the reduction of carboxyl groups (denoted as CF–OH). CF–OH could react with HNT–COOH easily to prepare HNT–COOH modified CFs. To form the covalent bonding with HNT–NH_2_, carboxyl groups on the surface of CF–COOH are needed to change to acyl chloride groups. Hence, CF–COOH was treated with the mixed solution of SOCl_2_ (100 mL) and DMF (5 mL) at 349 K for 24 h to get CF–COCl.

#### 2.3.2. Separate Grafting of HNT–NH_2_ and HNT–COOH onto CFs via Chemical Bonds

HNT–NH_2_/CF hierarchical reinforcements were prepared from the chemical reaction between HNT–NH_2_ and CF–COCl. HNT–NH_2_ (0.1 g) was firstly dispersed by ultrasonic in dried DMF (50 mL) for forming stable suspensions. Then, HNT–NH_2_ suspensions were mixed with CF–COCl (5 g) and reacted at 353 K for 24 h under nitrogen atmosphere. After modification, the obtained products (denoted as CF–HNT–NH_2_) were washed with DMF and acetone each many times and dried. To obtain HNT–COOH/CF hierarchical reinforcements via CF–OH bonded with HNT–COOH, once the suspension of HNT–COOH and DMF was ready, CF–OH (5 g), DMAP (0.01 g) and DCC (0.1 g) as catalysts were quickly added in HNT–COOH suspension and then reacted by stirring at 353 K for 24 h. Finally, CF–HNT–COOH was obtained after being washed by DMF and acetone each for several times and dried. The whole grafting processes are illustrated in Figure 1b.

### 2.4. Preparation of CF/MPSR Composites

CF/MPSR composite samples used for interfacial properties testing were prepared via the compression molding method. Appropriate untreated CF, CF–HNT–NH_2_ and CF–HNT–COOH were separately wrapped around a metal frame tightly. Then, the metal frame was soaked with MPSR solution to make MPSR saturate into the fibers. Subsequently, the unidirectional impregnating samples were obtained by being degassed with a vacuum pump until no bubbles or solvents came out of the samples. Finally, the composites were obtained via using a hot-pressing machine under the controlled curing schedule (atmospheric pressure for 1 h at 393 and 423 K, 20 MPa for 2 h at 473 K, and 20 MPa for 4 h at 523 K in sequence). The fiber contents in composites were about 70 mass%, and the dimensions of the measured cured samples were about 2 mm × 20 mm × 6 mm adopted for interfacial strength and hydrothermal aging resistance test.

### 2.5. Characterization Techniques

FTIR (Nicolet, Nexus670, Glendale, WI, USA) was used to examine chemical elements of untreated and modified HNTs. The testing specimens were characterized with 2 cm^−1^ resolution and 64 scans in the wavenumber range of 400–4000 cm^−1^. Surface composition and functional groups of different HNTs and the fibers before and after modification were also characterized by XPS (ESCALAB 220i-XL, VG, UK) with the monochromatic Al Ka source of 1486.6 eV at a base pressure of 2 × 10^−9^ mbar.

TGA (TA Instruments, Q50, New Castle, DE, USA) was used to examine thermal stability of HNTs, HNT–NH_2_ and HNT–COOH. The samples of 5–20 mg were added in aluminum pans, and heated from 30 to 800 °C in nitrogen atmosphere with the heating rate of 5 °C/min.

Surface characteristics of CFs before and after grafting and the cracked sections of MPSR composites reinforced with different CFs were studied by SEM (Quanta 200FEG, Hitachi Instrument, Inc., Tokyo, Japan). All testing samples were coated with thin gold layers via gold sputtering technique to increase fiber conductivity and obtain stable and clear images.

The changes of dynamic contact angles and surface energy analysis were carried out using a dynamic contact angle meter (DCAT21, Data Physics Instruments, Filderstadt, Germany), which were used to evaluate the wetting performance of untreated and HNTs modified CFs, using nonpolar diiodomethane and polar deionized water as the testing liquids. The surface free energy, and its dispersive as well as polar components of different CFs were obtained from the following equations:

(1) $$\gamma_{l}(1+\cos\theta )=2{\left(\gamma_{l}^{p}\gamma_{f}^{p}\right)}^{1/2}+2{\left(\gamma_{l}^{d}\gamma_{f}^{d}\right)}^{1/2}\;$$

(2) $$\gamma_{f}=\gamma_{f}^{p}+\gamma_{f}^{d}\;$$

where θ and $\gamma_{l}$ represent the dynamic contact angle and surface tension of testing liquids, respectively; $\gamma_{l}^{p}$ is the polar component; and $\gamma_{l}^{d}$ represents the dispersive component.

ILSS of untreated and modified composites were examined using a universal testing machine (WD-1, Changchun, China) based on a three-point short-beam bending testing method. Composites ILSS values could be calculated from:

(3) $$ILSS=\frac{3Pb}{4bh}\;$$

where *b* and *h* represent the width (mm) and thickness (mm) of the testing samples, and *P_b_* is the maximum breaking load (N).

Anti-hydrothermal aging experiments were carried out by adding CF/MPSR composites to boiling water at 373 K for 48 h to examine the effects of different functionalized HNT modifications on the hydrothermal aging resistance of the resulting composites. Afterwards, the hydrothermal aging resistance was characterized by tracing the changes of ILSS results.

## 3. Results

### 3.1. Surface Composition and Groups of Functionalized HNTs and CFs

**Figure 2:**
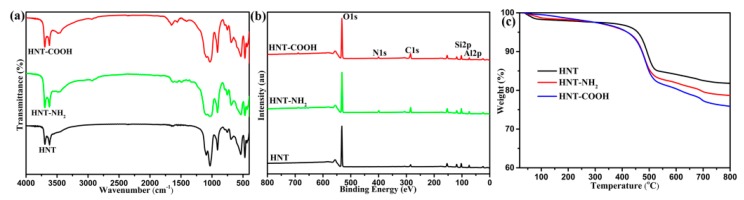
(**a**) FTIR spectra; (**b**) XPS spectra; and (**c**) TGA curves of untreated and modified HNTs.

Figure 2 shows FTIR spectra (Figure 2a), XPS spectra (Figure 2b) and TG curves (Figure 2c) of HNTs, HNT–NH_2_ and HNT–COOH. As for the FTIR spectrum of HNTs, the strong characteristic bands at about 3622 and 3698 cm^−1^ correspond to the Al–OH bonds stretching vibration, and the band at 907 cm^−1^ is related to the Al–OH bonds bending vibration onto the internal surface of HNTs [31]. The bands at round 1110 and 1033 cm^−1^ are related to the Si–O–Si bonds stretching vibration and asymmetric stretching vibration, respectively. In contrast to raw HNTs, HNT–NH_2_ shows a new characteristic band at round 2934 cm^−1^ corresponding to the stretching vibration of C–H bonds. Besides, the broad peaks in the range of 1600–1200 cm^−1^ are related to the stretching or bending vibrations of C–H, N–H and C–N bonds. These observed bonds arising from the structure of APS molecules indicate the success grafting of APS onto HNTs surface. For HNT–COOH, a significant band corresponding to the vibrations of carboxylic acids and secondary amides has been observed, and the peak is broad, while the peak intensity is fairly weak owing to few carboxylic acid and secondary amide groups and the existing hydrogen bonding interactions. More importantly, the additional peaks at about 1690 and 1570 cm^−1^ are due to C=O stretching vibrations of the carboxylic acid and amide, respectively [20]. These characteristic bands strongly verify the successful modification of HNTs from hydroxyls to carboxyl groups. As shown in Figure 2b, the XPS spectrum of HNTs is mainly composed of many peaks (O1s, Si2s, Si2p, and Al2p), which are consistent with chemical composition of HNTs mentioned in the literature [31]. Compared to HNTs, a new peak of N1s (401 eV) has been observed on HNT–NH_2_ XPS spectra arising from the element of APS. The peak intensity of C1s of HNT–COOH is stronger than that of HNT–NH_2_. The above FTIR and XPS results confirm that APS and succinic anhydride have been successfully grafted onto HNTs surface. Figure 2c presents TG curves of HNTs, HNT–NH_2_ and HNT–COOH. HNTs have a weight loss below 200 °C, which can be likely ascribed to the desorption of water. A second weight loss occurred from 450 to 550 °C owing to the pyrolysis of Al–OH functional groups on HNTs structure [31]. However, compared with TGA curves of HNTs, the higher weight loss of HNT–NH_2_ and HNT–COOH occurred between 200 °C and 450 °C because of the pyrolysis of the introduced organic silane onto modified HNTs after modification. A similar conclusion was also drawn by Zhu [32]. In other words, the weight loss of pristine HNTs is 18.3%, whereas the weight losses of HNT–NH_2_ and HNT–COOH reach 21.3 and 24.4%, respectively. TG results are consistent with the analysis of the above FTIR and XPS results, confirming the successful preparation of HNT–NH_2_ and HNT–COOH.

**Figure 3:**
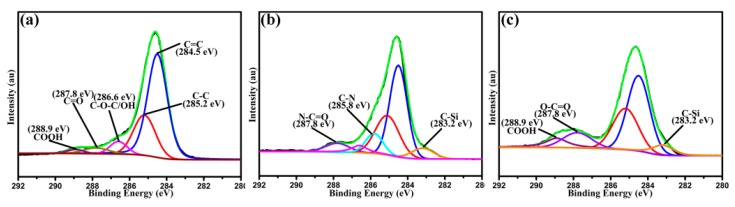
C1s high-resolution XPS peak-fitting curves of: (**a**) untreated CF; (**b**) CF–HNT–NH_2_; and (**c**) CF–HNT–COOH.

XPS was also used to characterize chemical composition and groups of different CFs for confirming the success of chemical modification. XPS C1s spectra of untreated CF, CF–HNT–NH_2_ and CF–HNT–COOH are presented in Figure 3. For untreated CF (Figure 3a), the XPS spectrum has been decomposed into five characteristic peaks (C=C, 284.5 eV; C–C, 285.2 eV; C–O, 286.6 eV; C=O, 287.8 eV; COOH, and 288.9 eV) [33]. As for the XPS spectrum of CF–HNT–NH_2_ (Figure 3b), many significant peaks have been detected. Two new peaks arising from C–Si (283.1 eV) and C–N (285.7 eV) appear, which may be caused by the introduced HNT–NH_2_ structure. Moreover, the presence of N–C=O with the bonding energies of about 287.8 eV further indicates that acyl chloride groups onto fiber surface have already reacted with amino groups of HNT–NH_2_. Hence, HNT–NH_2_ nanoparticles have been boned with the surface of CFs chemically. As seen from CF–HNT–COOH (Figure 3c), CF–OH was chemically modified with NHT–COOH, and introduced massive residual carboxyl groups onto CF–HNT–COOH surface. CF–HNT–COOH shows the sharp enhancement in the content of COOH peak and the obvious decrease of C–O content. In addition, the existences of the new peak O–C=O and C–Si are responsible for the success of chemical modification for introducing HNT–COOH onto fiber surface. The introduced amino and carboxyl groups help to change fiber inert surface to polar active one, which can improve fiber surface energy and wettability for significantly enhancing interfacial adhesion and mechanical properties of composites.

### 3.2. Surface Microstructures of CFs

**Figure 4:**
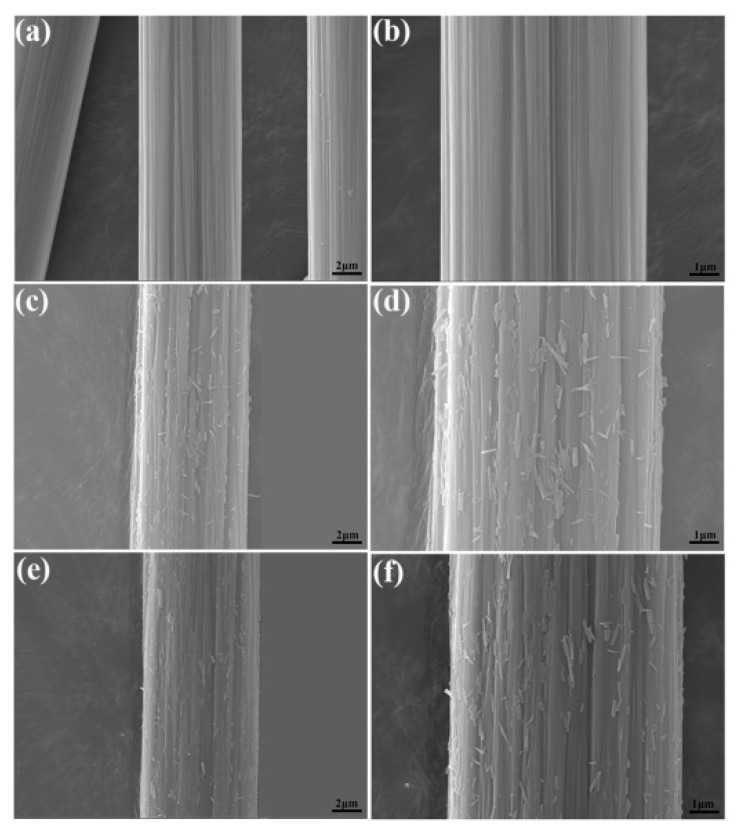
SEM images of different CF surfaces: (**a**,**b**) untreated CF, (**c**,**d**) CF–HNT–NH_2_; and (**e**,**f**) CF–HNT–COOH.

Figure 4 shows surface morphologies for CFs before and after HNTs modification. For untreated CF (Figure 4a,b), the fiber has a smooth and flat surface, and a few narrow parallel grooves are observed. In contrast, surface morphologies of CF–HNT–NH_2_ (Figure 4c,d) and CF–HNT–COOH (Figure 4e,f) change dramatically after modification, which increase fiber surface roughness significantly because of the uniform coverage of the fibers surface with grafted HNTs. Both have similar surfaces with uniform distributions of functionalized HNTs at different angles onto the surface and grooves of CFs, which make HNTs modified CFs appear as branched fibers for forming new hierarchical reinforcements. The introduced HNT–NH_2_ and HNT–COOH onto the fiber surface, inserting into the matrix resin, can connect the fibers and matrix resin tightly for increasing interfacial adhesion and mechanical properties of CF composites via increasing surface roughness to provide good mechanical interlocking.

### 3.3. Surface Wettability Analysis of CFs

The changes in chemical activity and surface topography of CFs affect surface energy (γ) of CFs. A high *γ* can change the wettability and compatibility between CFs and matrix resin. Hence, to study effects of HNT–NH_2_ and HNT–COOH modification on fiber surface energy and wettability, advancing contact angle (*θ*) and γ of untreated CF, CF–HNT–NH_2_ and CF–HNT–COOH are evaluated and listed in Table 1. The water contact angle (*θ*_water_) and diiodomethane angle (*θ*_diiodomethane_) of untreated CF are 78.5° and 58.9°, respectively. Therefore, *γ* of untreated CF is only 35.87 mN·m^−1^ (the dispersion component (γ^d^), 31.91 mN·m^−1^; and the polar component (γ^p^), 12.51 mN·m^−1^). However, compared with that of untreated CF, HNT–NH_2_ grafting and HNT–COOH grafting show remarkably decreased contact angles and sharply enhance fiber surface energy. *θ*_water_ of CF–HNT–NH_2_ and CF–HNT–COOH decreased to 44.28° and 42.95°. Similarly, *θ*_diiodomethane_ decreased to 40.06° for CF–HNT–NH_2_ and 38.77° for CF–HNT–COOH. As a result, *γ* of CF–HNT–NH_2_ and CF–HNT–COOH showed remarkable enhancements of 70.17% and 73.21% after two different functionalized HNTs grafting. The increased γ^p^ and γ^d^ can be related to the introduction of massive amino or carboxyl polar groups and the improvement of surface roughness of CFs grafted with HNT–NH_2_ and HNT–COOH, respectively. Noteworthy, CF–HNT–NH_2_ and CF–HNT–COOH have similar surface energy and wettability owing to the equal enhancements of chemical polar and surface roughness. As a result, the higher surface energy for the two prepared new hierarchical reinforcements helps to increase the wettability between HNTs modified CFs and MPSR, and then improved composites interfacial properties effectively.

### 3.4. Interfacial Property Testing of Composites

**Figure 5:**
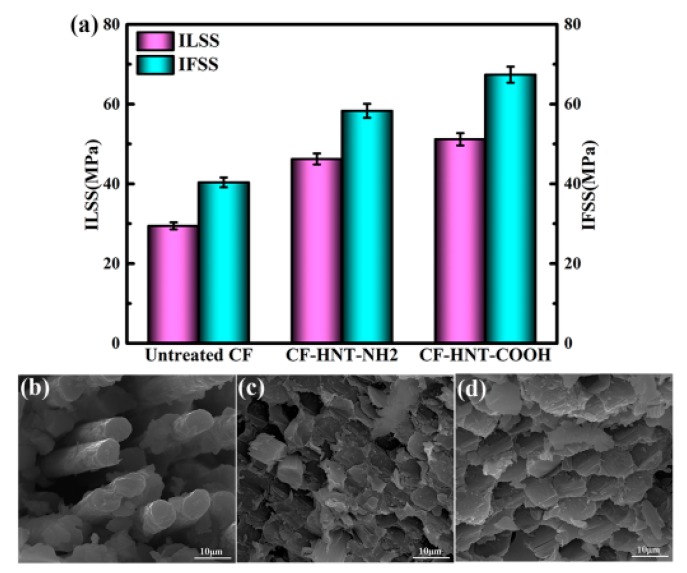
(**a**) ILSS and IFSS results of composites; and SEM morphologies of the fracture surface of MPSR composites reinforced with: (**b**) untreated CF; (**c**) CF–HNT–NH_2_; and (**d**) CF–HNT–COOH.

ILSS and IFSS testing results of MPSR composites reinforced with untreated CF, CF–HNT–NH_2_ and CF–HNT–COOH are presented in Figure 5a. ILSS and IFSS of untreated CF composites are only 29.47 and 40.37 MPa, which is related to fiber smooth and inert surface without providing a good compatibility with MPSR. After grafting, HNT–NH_2_ and HNT–COOH sharply enhance interfacial properties of composites because HNTs acting as an anchor stick into matrix resin to locally stiffen at the interface region for improving interface quality. For CF–HNT–NH_2_ composites, the ILSS and IFSS values enhanced to 46.23 and 58.31 MPa compared to those of untreated CF composites, which might be due to the improved interfacial wettability by the introduction of many amino groups as well as the formation of strong mechanical interlocking via the enhanced surface roughness caused by HNT–NH_2_ modified onto the fiber surface. After being grafted by HNT–COOH and CF–HNT–COOH, composites have the highest values of ILSS (51.19 MPa) and ILSS (67.38 MPa), which give rise to 73.70% and 66.91% enhancement in comparison with untreated CF composites, and 10.73% and 15.55% enhancement compared to CF–HNT–NH_2_ composites. The significant increases in ILSS and IFSS values with respect to CF–HNT–COOH composites can be mainly ascribed to the high compatibility and reactive activity of CFs by introduced massive carboxyl groups onto fiber surface. That is to say, the increased degree of interfacial properties directly correlates with the introduced active groups by functionalized HNTs modification. The formed chemical bonds between CFs and matrix resin make a critical contribution to the improvement of interfacial properties of composites. Combining the contrast of interfacial strength of CF–HNT–NH_2_ and CF–HNT–COOH composites with a wide range of nanomaterials modification (Supplementary Material, Table S1), the functionalized HNTs grafting strategy is comparable with CFs modified by other nanomaterials, and the enhanced effect on composites interfacial strength is superior to those of many CFs composites. Therefore, HNTs, as environment-friendly and cost-effective natural nanofillers, can be regarded as a commendable alternative to enhance the quality of fiber–matrix interface and mechanical properties of the resulting composites.

To fully study the interfacial enhancing mechanisms of MPSR composites reinforced with different fibers, composites fracture surfaces after ILSS testing were examined by SEM, as presented in Figure 5b–d. Many big holes exist in the cracked sections of untreated CF composites owing to massive the pulled-out fibers (Figure 5b), confirming the weak interfacial adhesion. After being grafted by HNT–NH_2_ (Figure 5c), the interfacial strength between CF–HNT–NH_2_ and MPSR is improved greatly. Massive resin fragments are scattered onto composite fracture surfaces with few holes and pulled-out CFs. However, some CFs remain detached from matrix resin with existing slight breakage of fibers. For CF–HNT–COOH composites (Figure 5d), a favorable and desired fracture surface without pulled-out fibers and fracture steps has been observed, which indicates the sharp improvement of interfacial adhesion and properties of composites via fiber surface grafting of HNT–COOH.

**Figure 6:**
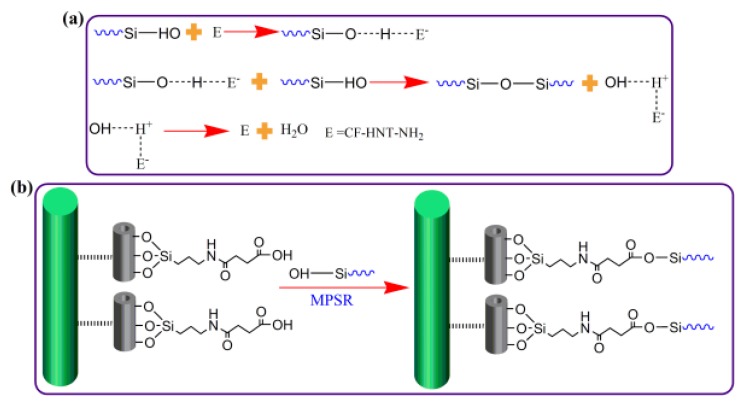
Schematic representation of the interfacial reaction of: (**a**) CF–HNT–NH_2_ composites; and (**b**) CF–HNT–COOH composites.

Figure 6 shows schematic illustration of the interfacial reaction of CF–HNT–NH_2_ and CF–HNT–COOH composites. For two functionalized HNTs modified CF composites, HNT–NH_2_ and HNT–COOH grafting provide similar wettability and mechanical interlocking caused by the obtained fiber surface energy and roughness according to DCA and SEM testing. However, the formation of interfacial reinforcing mechanisms is completely different. For CF–HNT–NH_2_ composites (Figure 6a), the introduced amino groups of CFs by HNT–NH_2_ grafting using as the basic catalysts for matrix resin cannot react with MPSR resin during the preparation process of composites, but activate the hydroxyl groups of MPSR matrix for accelerating the cross-linking process. This is to say, the introduced HNT–NH_2_ can only produce sufficient mechanical interlocking for improving interfacial compatibility and adhesion between CF–HNT–NH_2_ and MPSR. For CF–HNT–COOH/MPSR composites (Figure 6b), the presence of carboxyl groups onto the CF–HNT–COOH has the high reaction activity with MPSR. The introduced chemical bonding leads to a significant improvement in the stress transfer from MPSR to HNT–COOH modified hierarchical reinforcing structure, and this is a crucial factor for interfacial improvement. Hence, the chemical bonds combined with sufficient nanosized mechanical interlocking at the interface region via the high strength of the HNT-network modified onto the CF surface inhibit the shear flow via the interface effectively, leading to the best interfacial adhesion of composites.

### 3.5. Hydrothermal Aging Resistance Testing of Composites

**Figure 7:**
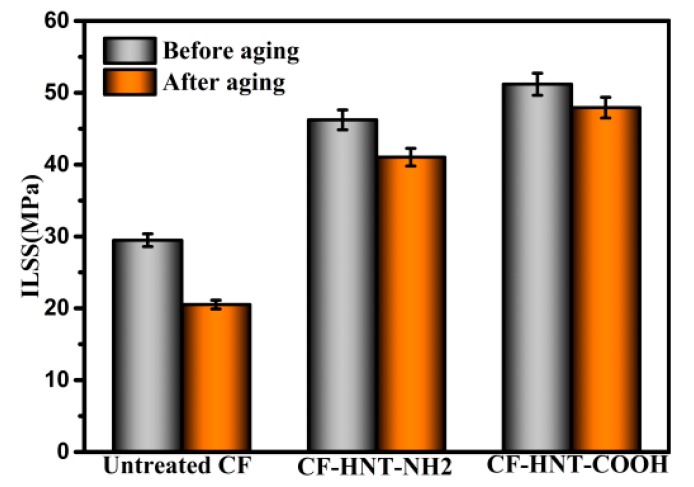
ILSS of composites before and after hydrothermal aging treatment.

The hydrothermal aging experiments give CF/MPSR composites direct insights to the potential applications in the harsh environment with high humidity. ILSS value retentions of untreated and modified CF composites after aging are shown in Figure 7. After aging, the ILSS values of untreated CF/MPSR composites decline sharply, while ILSS values of MPSR composites reinforced with CF–HNT–NH_2_ and CF–HNT–COOH declines slower. ILSS values of untreated CF reinforcing MPSR composites decreased from 29.47 MPa without aging to 20.52 MPa, with ILSS retention ratios of 69.63%. However, ILSS retention ratios of MPSR composites reinforced with CF–HNT–NH_2_ and CF–HNT–COOH are 88.75% and 93.61%, respectively, confirming that HNT–NH_2_ or HNT–COOH functionalization can enhance hydrothermal aging resistance of the resulting composites. A poor quality of fiber–matrix interface containing more microcracks and drawbacks is penetrated easily via water molecules because of the obvious difference in coefficient of thermal expansion, which can form the stress concentration at the interfacial region and thus destroy the interface, resulting in a poor hydrothermal aging resistance of untreated CF composites. The introduced HNT–NH_2_ or HNT-COOH can strengthen interfacial adhesion between CFs and MPSR and decrease the numbers of microcracks and defects at the interfacial region, which reduce water absorption and protect the interface effectively compared with untreated composites. In addition, compared with CF–HNT–NH_2_ composites, CF–HNT–COOH composites show higher hydrothermal aging resistance. This might be related to the better quality of HNT–COOH interface, which would require stronger acid/base and more energy to destroy.

## 4. Conclusions

In this study, to improve interfacial properties and anti-hydrothermal aging behaviors of CF/MPSR composites, the chemical grafting of different functionalized HNTs as natural and low-cost nanomaterials onto CFs was reported. Characterization results confirmed the successful modification of functionalized HNTs, and HNT–NH_2_ or HNT–COOH was modified onto the surface of CFs uniformly. HNT–NH_2_ or HNT–COOH modification improved surface wettability significantly through the introduced amino or carboxyl polar groups as well as increased mechanical interlocking between CFs and MPSR obviously via the enhanced surface roughness, leading to the sharp improvement in interfacial strength of composites. Particularly, MPSR composites reinforced with CF–HNT–COOH have the best interfacial properties with ILSS value of 51.19 MPa and IFSS value of 67.38 MPa compared with those of CF–HNT–NH_2_ composites (ILSS, 46.23 MPa; and IFSS, 58.31 MPa). The improved mechanical interlocking and the formed chemical bonds between CF–HNT–COOH and MPSR through HNT–COOH nanoparticle modification were the main contributors for these highest enhancements. In addition, the introduced HNT–NH_2_ or HNT–COOH at the interface increased the composites’ hydrothermal aging resistance effectively.

## Figures and Tables

**Table 1:** Contact angles and surface energy of different CFs.

Samples	Contact Angles (°)		Surface Energy (mN·m^−1^)		
	*θ*_water_	*θ*_diiodomethane_	γ^d^	γ^p^	γ
Untreated CF	78.50	58.90	29.21	6.66	35.87
CF–HNT–NH_2_	44.28	40.06	21.46	39.58	61.04
CF–HNT–COOH	42.95	38.77	21.91	40.22	62.13

## Supplementary Materials

The following are available online at http://www.mdpi.com/2073-4360/10/10/1171/s1, Table S1: Comparison of interfacial properties among different nanomaterials modified carbon fibers composites.
