# Supplementary material for: Grafting Halloysite Nanotubes with Amino or Carboxyl Groups onto Carbon Fiber Surface for Excellent Interfacial Properties of Silicone Resin Composites
Source: Polymers (Basel). 2018 Oct 22;10(10):1171. doi: 10.3390/polym10101171 (PMC6403793; doi:10.3390/polym10101171)
Supplement: Supplementary file 1 [file polymers-10-01171-s001.pdf]

Supplementary Materials: Grafting Halloysite Nanotubes with Amino or Carboxyl Groups onto Carbon Fiber Surface for Excellent Interfacial Properties of Silicone Resin Composites

Xiandong Zhang and Guangshun Wu

Table S1. Comparison of interfacial properties among different nanomaterials modified carbon fibers composites.

Nanofiller	Resin Type	Imodification Method	IFSS Improvement (%)	Ref.
Halloysite nanotubes with carboxyl groups	Silocone resin	Chemical grafting	66.91%	This work
Carboxylic acid- functionalized carbon nanotubes	Epoxy resin	Chemical grafting	41.55%	[1]
Oxidized multiwall carbon nanotubes	Epoxy resin	Electrophoretic deposition	33.3%	[2]
Acyl chloride functionalized graphene oxide	Epoxy resin	Chemical grafting	46.53%	[3]
Graphene oxide	Epoxy resin	Polymer sizing	70.9%	[4]
Halloysite nanotubes	Epoxy resin	Dip coating	61%	[5]
Amino-functionalized nanoclay	Epoxy resin	Chemical grafting	33%	[6]
octa(aminophenyl) polyhedral oligomeric silsesquioxane	Silocone resin	Chemical grafting	47.83%	[7]
Nano-sized titanium dioxide	Epoxy resin	thiol–ene click chemistry	78.05%	[8]
Aramid nanofibers	Epoxy resin	Electrophoretic deposition	34.9%	[9]
Silanized silica nanoparticles	Silocone resin	Chemical grafting	40.92%	[10]
Titanium dioxide nanowires	Epoxy resin	A hydrothermal growth method	44.7%	[11]

1. Li, Y.; Li, Y.; Ding, Y.; Peng, Q.; Wang, C.; Wang, R.; Sritharan, T.; He, X.; Du, S. Tuning the interfacial property of hierarchical composites by changing the grafting density of carbon nanotube using 1,3-propodiamine. *Compos. Sci. Technol.* **2013**, *85*, 36–42.
2. Sui, X.; Shi, J.; Yao, H.; Xu, Z.; Chen, L.; Li, X.; Ma, M.; Kuang, L.; Fu, H.; Deng, H. Interfacial and fatigue-resistant synergetic enhancement of carbon fiber/epoxy hierarchical composites via an electrophoresis deposited carbon nanotube-toughened transition layer. *Compos. Part A* **2017**, *92*, 134–144.
3. Gao, B.; Zhang, R.; He, M.; Sun, L.; Wang, C.; Liu, L.; Zhao, L.; Cui, H.; Cao, A. Effect of a multiscalereinforcement by carbon fiber surface treatment with graphene oxide/carbon nanotubes on the mechanical properties of reinforced carbon/carbon composites. *Compos. Part A* **2016**, *90*, 433–440.
4. Zhang, X.; Fan, X.; Yan, C.; Li, H.; Zhu, Y.; Li, X.; Yu, L. Interfacial microstructure and properties of carbon fiber composites modified with graphene oxide. *ACS Appl. Mater. Inter.* **2012**, *4*, 1543–1552.
5. Jäger, M.; Zabihi, O.; Ahmadi, M.; Li, Q.; Depalmeanar, A.; Naebe, M. Nano-enhanced interface in carbon fibre polymer composite using halloysite nanotubes. *Compos. Part A* **2018**, *109*, 115–123.
6. Zabihi, O.; Ahmadi, M.; Li, Q.; Shafei, S.; Huson, M.G.; Naebe, M. Carbon fibre surface modification using functionalized nanoclay: A hierarchical interphase for fibre-reinforced polymer composites. *Compos. Sci. Technol.* **2017**, *148*, 49–58.
7. Wu, G.; Ma, L.; Jiang, H.; Liu, L.; Directly grafting octa(aminophenyl) polyhedral oligomeric silsesquioxane onto carbon fibers for superior interfacial strength and hydrothermal aging resistance of silicone resin composites. *Constr. Build. Mater.* **2017**, *157*, 1040–1046.
8. Xiong, L.; Zhan, F.; Liang, H.; Chen, L.; Lan, D. Chemical grafting of nano-TiO_2 onto carbon fiber via thiol–ene click chemistry and its effect on the interfacial and mechanical properties of carbon fiber/epoxy composites. *J. Mater. Sci.* **2017**, *53*, 2594–2603.
9. Lee, J.U.; Park, B.; Kim, B.-S.; Bae, D.-R.; Lee, W. Electrophoretic deposition of aramid nanofibers on carbon fibers for highly enhanced interfacial adhesion at low content. *Compos. Part A* **2016**, *84*, 482–489.
10. Wu, G.; Ma, L.; Jiang, H.; Liu, L.; Huang, Y. Improving the interfacial strength of silicone resin composites by chemically grafting silica nanoparticles on carbon fiber. *Compos. Sci. Technol.* **2017**, *153*, 160–167.
11. Ma, L.; Li, N.; Wu, G.; Song, G.; Li, X.; Han, P.; Wang, G.; Huang, Y. Interfacial enhancement of carbon fiber composites by growing TiO_2 nanowires onto amine-based functionalized carbon fiber surface in supercritical water. *Appl. Surf. Sci.* **2018**, *433*, 560–567.
